# Supplementary material for: Stop codon context influences genome-wide stimulation of termination codon readthrough by aminoglycosides
Source: eLife. 2020 Jan 23;9:e52611. doi: 10.7554/eLife.52611 (PMC7089771; doi:10.7554/eLife.52611)
Supplement: Supplementary file 1. [file elife-52611-supp1.docx]

| **Key Resources Table** | | | | |
| --- | --- | --- | --- | --- |
| **Reagent type (species) or resource** | **Designation** | **Source or reference** | **Identifiers** | **Additional information** |
| cell line (*Homo-sapiens*) | HEK293T | ATCC | CRL-3216; RRID:CVCL_0063 |  |
| cell line (*Homo-sapiens*) | Calu-6 | ATCC | HTB-56; RRID:CVCL_0236 |  |
| antibody | anti-TP53 (Mouse monoclonal) | Santa Cruz Biotechnology | DO-1;  Cat# sc-126; RRID:AB_628082 | WB (1:100) |
| antibody | Goat-anti-mouse IgG secondary HRP conjugate | Thermo Fisher | Cat# 32430; RRID:AB_1185566 | WB (1:1000) |
| antibody | anti-β-Actin-HRP (rabbit monoclonal) | Cell Signaling Technologies | 13E5;  Cat# 5125; RRID:AB_1903890 | WB (1:5000) |
| recombinant DNA reagent | pcDNA5-dual-FLuc-NLuc  (plasmid) | This paper | pJRW002 | Full-length  NLuc |
| recombinant DNA reagent | pcDNA5-dual-FLuc-NLuc  (plasmid) | This paper | pJRW003 | NLuc-R154X-UGA |
| recombinant DNA reagent | pcDNA5-dual-FLuc-NLuc  (plasmid) | This paper | pJRW004 | NLuc-R154X-UAA |
| recombinant DNA reagent | pcDNA5-dual-FLuc-NLuc  (plasmid) | This paper | pJRW005 | NLuc-R154X-UAG |
| recombinant DNA reagent | pcDNA5-dual-FLuc-NLuc  (plasmid) | This paper | pJRW006 | NLuc-W12X-UAA |
| recombinant DNA reagent | pcDNA5-dual-FLuc-NLuc  (plasmid) | This paper | pJRW007 | NLuc-W12X-UAG |
| recombinant DNA reagent | pcDNA5-dual-FLuc-NLuc  (plasmid) | This paper | pJRW008 | NLuc-W12X-UGA |
| recombinant DNA reagent | pcDNA5-dual-FLuc-NLuc  (plasmid) | This paper | pJRW009 | NLuc-V40X-UAA |
| recombinant DNA reagent | pcDNA5-dual-FLuc-NLuc  (plasmid) | This paper | pJRW010 | NLuc-V40X-UAG |
| recombinant DNA reagent | pcDNA5-dual-FLuc-NLuc  (plasmid) | This paper | pJRW011 | NLuc-V40X-UGA |
| recombinant DNA reagent | pcDNA5-dual-FLuc-NLuc  (plasmid) | This paper | pJRW012 | NLuc-E51X-UAA |
| recombinant DNA reagent | pcDNA5-dual-FLuc-NLuc  (plasmid) | This paper | pJRW013 | NLuc-E51X-UAG |
| recombinant DNA reagent | pcDNA5-dual-FLuc-NLuc  (plasmid) | This paper | pJRW014 | NLuc-E51X-UGA |
| recombinant DNA reagent | pcDNA5-dual-FLuc-NLuc  (plasmid) | This paper | pJRW015 | NLuc-H88X-UAA |
| recombinant DNA reagent | pcDNA5-dual-FLuc-NLuc  (plasmid) | This paper | pJRW016 | NLuc-H88X-UAG |
| recombinant DNA reagent | pcDNA5-dual-FLuc-NLuc  (plasmid) | This paper | pJRW017 | NLuc-H88X-UGA |
| recombinant DNA reagent | pcDNA5-dual-FLuc-NLuc  (plasmid) | This paper | pJRW018 | NLuc-G113X-UAA |
| recombinant DNA reagent | pcDNA5-dual-FLuc-NLuc  (plasmid) | This paper | pJRW019 | NLuc-G113X-UAG |
| recombinant DNA reagent | pcDNA5-dual-FLuc-NLuc  (plasmid) | This paper | pJRW020 | NLuc-G113X-UGA |
| recombinant DNA reagent | pcDNA5-dual-FLuc-NLuc  (plasmid) | This paper | pJRW021 | NLuc-W134X-UAA |
| recombinant DNA reagent | pcDNA5-dual-FLuc-NLuc  (plasmid) | This paper | pJRW022 | NLuc-W134X-UAG |
| recombinant DNA reagent | pcDNA5-dual-FLuc-NLuc  (plasmid) | This paper | pJRW023 | NLuc-W134X-UGA |
| chemical compound, drug | Gentamicin sulfate | Milipore Sigma | G1264 | powder |
| chemical compound, drug | Paromomycin sulfate | Milipore Sigma | P9297 | powder |
| chemical compound, drug | Neomycin trisulfate | Milipore Sigma | N6386 | powder |
| chemical compound, drug | Tobramycin | Milipore Sigma | T4014 | powder |
| chemical compound, drug | Amikacin sulfate | Milipore Sigma | A2324 | powder |
| chemical compound, drug | G418 sulfate | Thermo Fisher | 11811023 | powder |
| chemical compound, drug | Gentamicin | Thermo Fisher | 15750060 | solution |
| chemical compound, drug | G418 | Thermo Fisher | 10131035 | solution |
| chemical compound, drug | cycloheximide | Milipore Sigma | C1988 | powder |
| commercial assay or kit | Nano-Glo Dual-Luciferase Reporter Assay System | Promega | N1630 |  |
| commercial assay or kit | BCA Protein Assay | Thermo Fisher | 23225 |  |
| commercial assay or kit | Quant-iT RiboGreen RNA Assay | Thermo Fisher | R11490 |  |
| commercial assay or kit | miRNeasy mini kit | Qiagen | 217004 |  |
| commercial assay or kit | RNeasy mini kit | Qiagen | 74104 |  |
| commercial assay or kit | RiboZero Gold Human/Mouse/Rat | Illumina | MRZG12324 | Sadly this is now discontinued |
| commercial assay or kit | Bioanalyzer High Sensitivity DNA Analysis | Agilent | 5067-4626 |  |
| commercial assay or kit | TruSeq Stranded Total RNA Library Prep Gold | Illumina | 20020598 |  |
| peptide, recombinant protein | Turbo DNase | Thermo Fisher | AM2239 |  |
| peptide, recombinant protein | RNase I | Thermo  Fisher | AM2295 | Ambion – sourced |
| peptide, recombinant protein | SUPERase*In | Thermo Fisher | AM2696 |  |
| peptide, recombinant protein | T4 PNK | New England Biolabs | M0201L |  |
| peptide, recombinant protein | T4 RNA ligase 2 – truncated | New England Biolabs | M0242L |  |
| peptide, recombinant protein | Superscript III | Thermo Fisher | 18080044 |  |
| peptide, recombinant protein | CircLigase ssDNA Ligase | Lucigen | CL4115K |  |
| peptide, recombinant protein | Phusion high-fidelity polymerase | New England Biolabs | M0530L |  |
| software, algorithm | tally | Davis et al., 2013; DOI: 10.1016/j.ymeth.2013.06.027 | 15-065 |  |
| software, algorithm | seqtk | Heng Li. 2016; https://github.com/lh3/seqtk | 1.0-r31 |  |
| software, algorithm | skewer | Jiang et al., 2014; https://github.com/lh3/seqtk | 0.2.2 |  |
| software, algorithm | STAR | Dobin et al., 2013; 10.1093/bioinformatics/bts635 | STAR_2.5.3a_modified |  |
| software, algorithm | pigz | https://zlib.net/pigz/ | 2.3.1 |  |
| software, algorithm | samtools | https://github.com/samtools/samtools | 0.1.19-96b5f2294a |  |
| software, algorithm | kpLogo | Wu and Bartel., 2017; DOI: 10.1093/nar/gkx323 |  |  |
| software, algorithm | kentUtils | https://github.com/ENCODE-DCC/kentUtils |  |  |
| software, algorithm | Custom Software (Python 2.7 and R 3.4.3) | https://github.com/jrw24/G418_readthrough |  | Contains required scripts to reproduce all figures |
| sequence-based reagent | Preadenylated linker | Wu et al., 2019; DOI: 10.1016/J.MOLCEL.2018.12.009 | oBZ407 | rAppNNNNNNCACTCGGGCACCAAGGAC |
| sequence-based reagent | RT primer | Wu et al., 2019; DOI 10.1016/J.MOLCEL.2018.12.009 | oBZ408 | /5Phos/RNNNAGATCGGAAGAGCGTCGTG TAGGGAAAGAGTGTAGATCTCGGTGGTC GC/iSP18/TTCAGACGTGTGCTCTTCCGA TCTGTCCTTGGTGCCCGAGTG |
