# Supplementary material for: Stop codon context influences genome-wide stimulation of termination codon readthrough by aminoglycosides
Source: eLife. 2020 Jan 23;9:e52611. doi: 10.7554/eLife.52611 (PMC7089771; doi:10.7554/eLife.52611)
Supplement: Supplementary file 2. [file elife-52611-supp2.docx]

**Supplemental Table 2:** RefSeq Identifiers of sequences used for rRNA depletion

| rRNA gene | RefSeq ID |
| --- | --- |
| 28S | NR_145822.1 |
|  | NR_146148.1 |
|  | NR_146154.1 |
|  | NR_146118.1 |
|  | NR_003287.4 |
| 18S | NR_145820.1 |
|  | NR_146146.1 |
|  | NR_146152.1 |
|  | NR_146119.1 |
|  | NR_003286.4 |
| 5.8S | NR_145821.1 |
|  | NR_146147.1 |
|  | NR_146153.1 |
|  | NR_146120.1 |
|  | NR_003285.3 |
| 5S | NR_023363.1 |
|  | NR_023364.1 |
|  | NR_023365.1 |
|  | NR_023366.1 |
|  | NR_023367.1 |
|  | NR_023368.1 |
|  | NR_023369.1 |
|  | NR_023370.1 |
|  | NR_023371.1 |
|  | NR_023372.1 |
|  | NR_023373.1 |
|  | NR_023374.1 |
|  | NR_023375.1 |
|  | NR_023376.1 |
|  | NR_023377.1 |
|  | NR_023378.1 |
|  | NR_023379.1 |
